# Characterization of canola oil extracted by different methods using fluorescence spectroscopy

**DOI:** 10.1371/journal.pone.0208640

**Published:** 2018-12-17

**Authors:** M. Saleem, Naveed Ahmad

**Affiliations:** 1 Agri. & Biophotonics Division, National Institute of Lasers and Optronics (NILOP), Lehtrar road, Islamabad, Pakistan; 2 Department of Physics, Mirpur University of Science and Technology (MUST) Mirpur, Azad Kashmir, Pakistan; Universita degli Studi di Perugia, ITALY

## Abstract

The potential of fluorescence spectroscopy has been utilized for the characterization of three types of canola oil samples: the first type was obtained by dissolving its seeds in hexane solvent, the second by cold press method, and the third from eight commercial brands. Fluorescence spectra from all samples have been acquired by using excitation wavelengths from 280 to 420 nm with step of 10 nm to investigate their valuable ingredients. The emission bands at 375, 525 and 673 nm that represent vitamin E/beta-carotene and chlorophyll, are present only in canola oil samples extracted by chemical and cold press methods and absolutely absent from all commercial brands. The emission band at 440 nm appearing only in the commercial oil brands, is assigned to oxidized products of isomers of vitamin E and fatty acids. In addition, the effect of temperature on the canola oil extracted by cold press method has been investigated which showed that up to 180 ^o^C it does not lose much of its natural molecular composition. However, it showed a trend of thermal oxidation with rise of temperature.

## Introduction

Canola oil is extracted from rapeseed and consumed all over the world due to its valuable ingredients [1]. It has a low amount of saturated and a substantial amount of monounsaturated fats with roughly 2:1 mono to polyunsaturated fatty acids [2]. In general, it contains 61% oleic acid which is classified as a monounsaturated omega-9 fatty acid, 11% α-linolenic acid and 21% linoleic acid which are omega-3 and omega-6 polyunsaturated fatty acids, and 7% saturated fatty acids [3,4]. Canola oil is second to olive oil in oleic acid content and intermediate among other vegetable oils in polyunsaturated fatty acid (PUFA). It contains high level of PUFA comparing to olive and palm oil but lower level than corn, soybean, and sunflower oils [3]. In addition, it contains phytosterols, tocopherols, which are biologically active isomers of vitamin E [3,5], beta-carotenes and chlorophylls [3].

Canola oil is extracted by slightly heating the crushed canola seeds dissolved in the hexane solvent or by cold press method [4]. Finally, it is refined using water precipitation and organic acid to remove gums and free fatty acids, filtering to remove color, and deodorizing using steam distillation [4]. Refining methods largely remove vitamin E, carotenoids and chlorophylls during bleaching [6] and deodorization processes [7]. Refining process renders canola oil a hydrogenated mess of trans fatty acids and their consumption may lead to heart problems, blood platelet abnormalities, increased cancer risk and free radical damage [3].

Characterization of any edible oil is therefore very important because the problems related with its quality and adulteration [8,9] is very common across the world which is determined by its peroxide value, a most traditional and used parameter for measuring its deterioration [10,11]. Fluorescence spectroscopy, on the other hand, proved itself an on line, quick, non-invasive and reliable tool for the characterization of food [12]. It has been used extensively as a fingerprint optical technique for the quality assurance of edible oils [13–19].

In the present article, three types of canola oils have been spectroscopically investigated using fluorescence spectroscopy. It has been found that the commercially available canola oil brands do not contains vitamin E, beta-carotene and chlorophylls, which should be naturally present. In addition, the effect of temperature on the cold pressed canola oil has been investigated to find a safe temperature range for cooking and frying of food.

## Materials and methods

### Sample preparation

**Fig 1:**
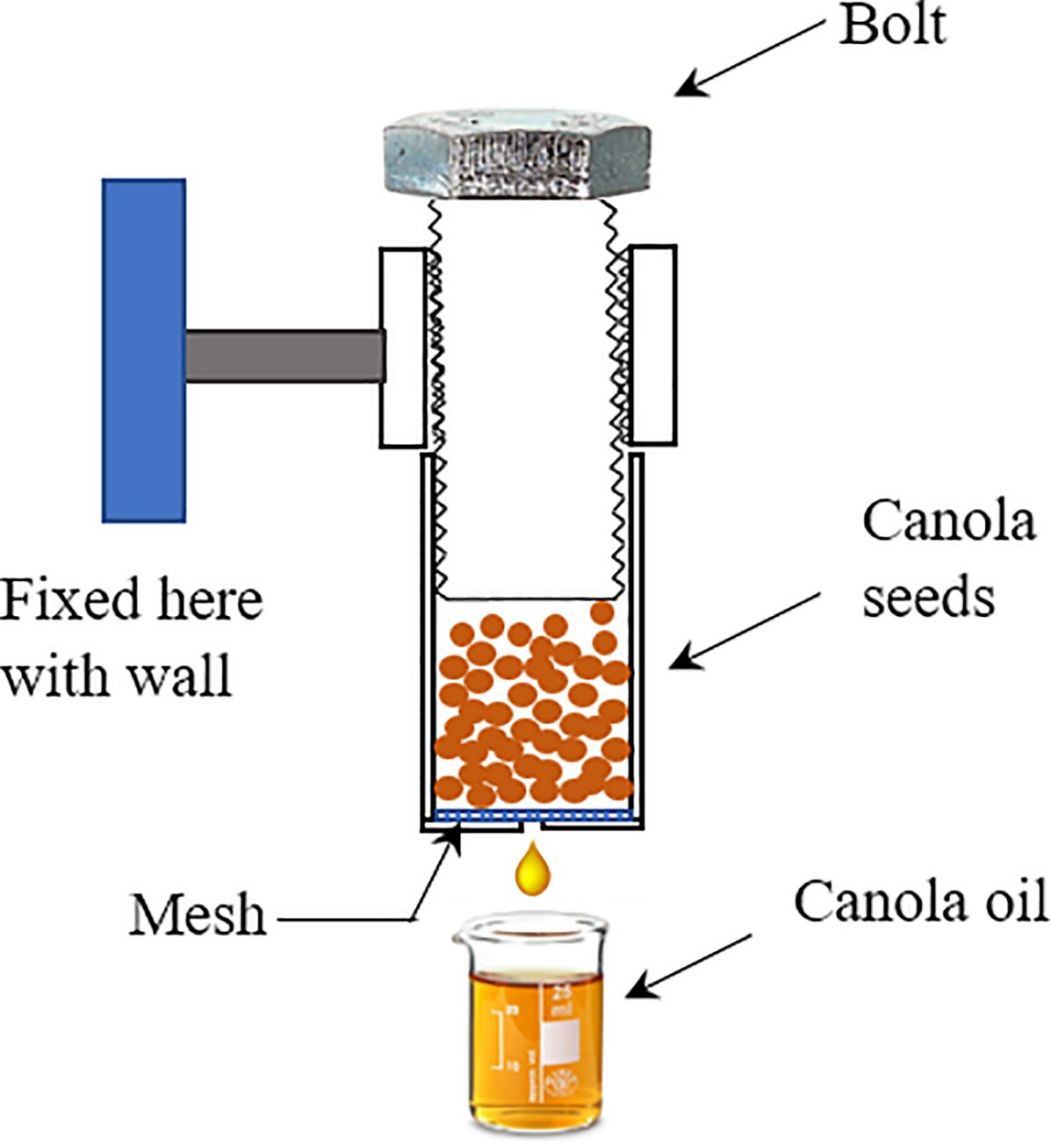
Schematic diagram of the cold press oil extraction setup.

First type of canola oil sample was extracted in the laboratory by dissolving crushed canola seeds in hexane solvent (Sigma-Aldrich). Canola seeds were crushed in mortar and pestle. The mixture was left for three days during which seeds dissolved completely in hexane. The mixture was then filtered through Whatman qualitative filter grade-1 (Sigma Aldrich) to get clean canola oil. It was further placed in a glass beaker with open lid at room temperature which varied from 25 to 35 ^o^C in summer at Islamabad for a week to evaporate hexane completely. The second type of oil sample was obtained by cold press mechanism in the laboratory. The clean canola seeds were poured in a stainless-steel cylinder which has a hole in the bottom, as shown in Fig 1. When bolt shown in Fig 1 is screwed down, it presses canola seeds slowly to extract oil which is collected in the beaker. The hole is covered with a stainless steel mesh to stop crushed residues coming out from the hole with oil. This extracted canola oil through cold press method may be called virgin oil.

**Fig 2:**
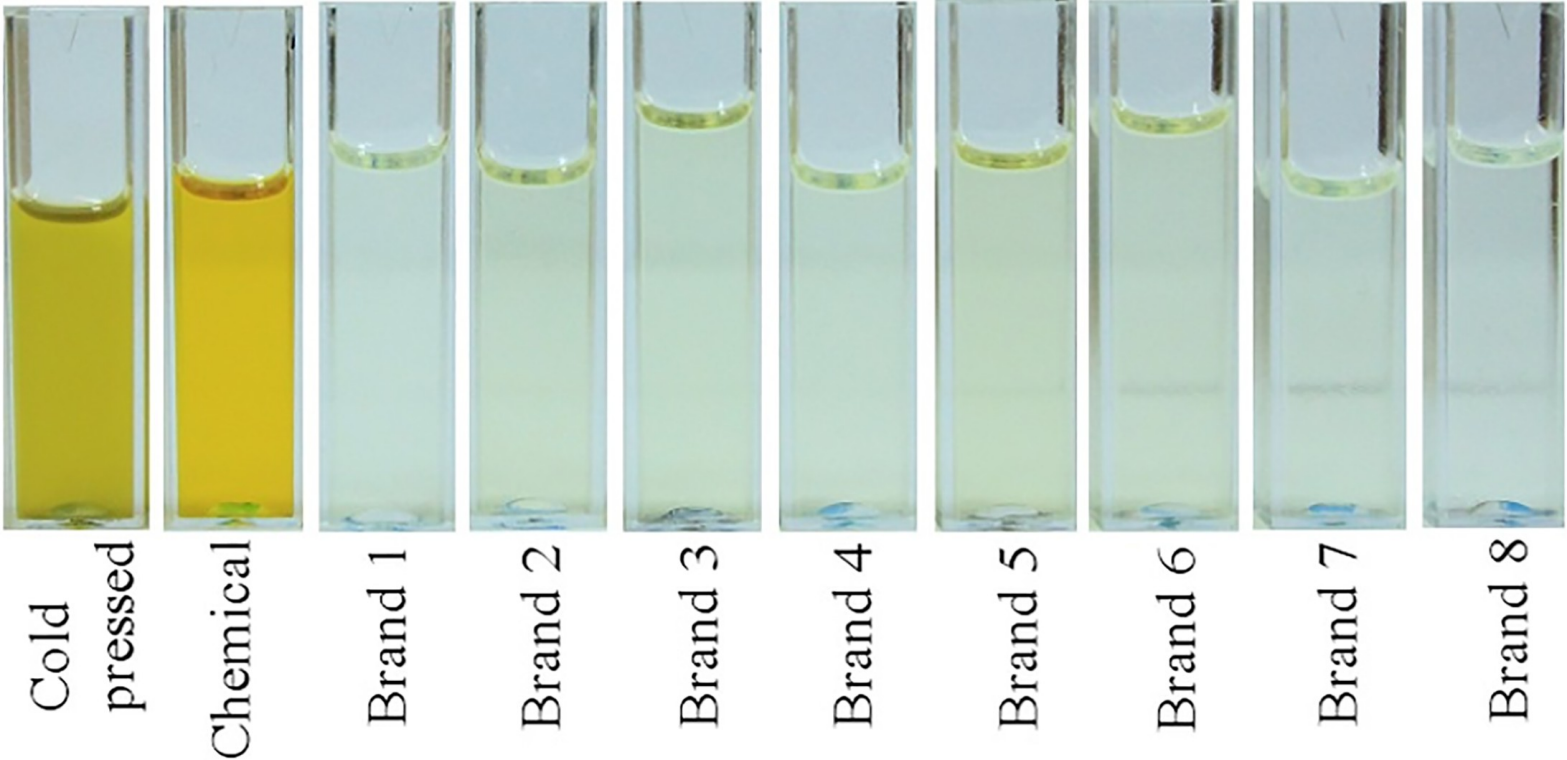
Visual display of cold pressed, chemically extracted and eight commercial canola brands purchased from local markets of Islamabad, Pakistan.

The third type of canola oil samples were taken from eight commercial canola brands purchased from the local markets of Islamabad, Pakistan. Three types of canola oil samples including cold pressed, chemically extracted and eight commercial brands, are displayed in Fig 2 for a nice visual comparison.

In subcontinent, a generally opted cooking method is described below. At the begging of cooking, an appropriate amount of oil, crushed onion, ginger and garlic are poured into a cooking pot which is heated by a gas burner or an electric stove until onion turns light brown. The temperature of mixture was monitored by a K-type thermocouple that measures temperature directly with an accuracy of ±2°C. It was found that temperature of mixture in cooking pot remains between 100 and 110 ^o^C and it took 5–10 minutes to turn whole mixture to light brown. The cooking time may depend upon amount of food stuff and supplied heat. At this stage, a small quantity of water was poured in the pot so that the above described mixture was just immersed, along with appropriate quantities of other food items including green/red chili, tomato, coriander and turmeric are also added. Then the whole mixture was cooked for 5 minutes by tightly fixing the lid of the pressure cooker. After 5 minutes, lid was opened and the mixture was further fried for 5–10 minutes until oil got itself separated from the mixture. The temperature of mixture remained around 100°C due to water content. When all mixture got fried, water content reduces which causes the temperature of mixture to increase up to 115°C. Further heating causes the mixture to burning. At this stage when oil got separated itself from the mixture, vegetable or meat are added and cooked. The whole procedure took around 20 minutes where temperature remains between 100 and 115°C.

By keeping in view the above described cooking method, heated samples of cold pressed canola oil have been prepared by heating them at the following temperatures of 100, 110, 120, 130, 150, 170, 180, 200 and 250°C in the electrical oven, by considering that the smoke point of canola oil is reported at 220–230°C [5] and 238°C [20]. In addition, a commercial brand-2 has been heated at different temperatures of 100, 150, 200 and 250°C for time duration of 30 minutes to investigate the effect of temperature on the evolution of oxidized products. Sample of vitamin E (Evion capsules, Merck Pvt. Ltd. Pakistan) has been purchased from the local market.

### Acquisition of fluorescence spectra

**Fig 3:**
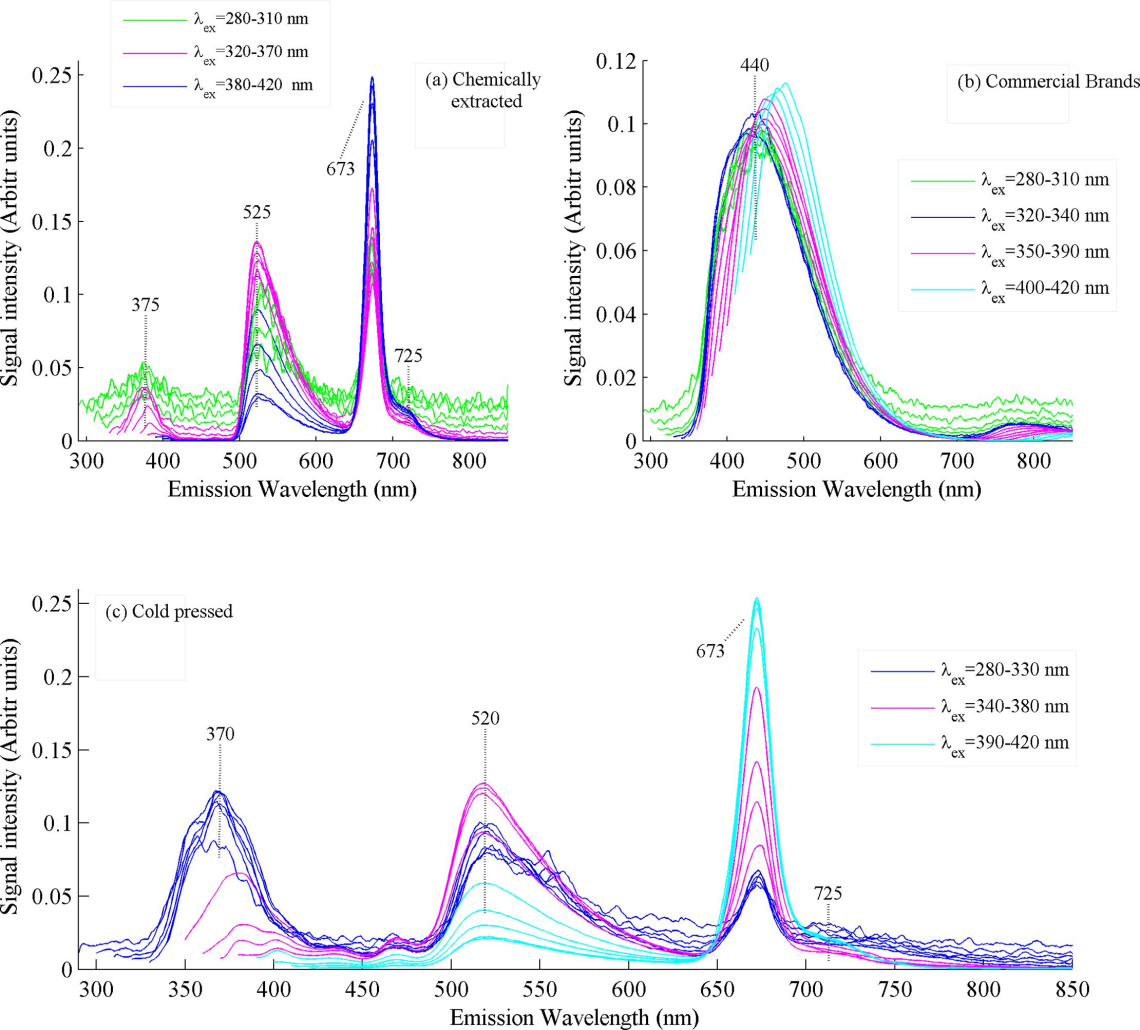
Fluorescence spectra of cold pressed, chemically extracted and commercial brands of canola oil samples using excitation wavelengths in the range 280–420 nm.

The fluorescence spectra of all canola oil samples using different exciting wavelengths in the range 280–420 nm have been acquired by a spectrofluorometer system FluoroMax-4 (Horiba Scientific, Jobin Yvon inc, USA). The system was controlled by a software fluoroEssence^TM^ operating within the Window’s environment. A continuous 150 W ozone free xenon arc lamp is used as the excitation source and the photomultiplier (R928P) was used as the detector. Fluorescence spectra of all samples were acquired by pouring samples in a cuvette through right-angle geometry with an acquisition time of 1 sec. Spectroscopic analysis of the emission spectra shown in Fig 3 put in evidence that the maxima of the emission bands appeared at the two exciting wavelengths of 330 and 410 nm, which were selected as excitation sources for further recording the spectra and statistical analysis. Both selected wavelengths produced maximum spectral information about samples.

### Preprocessing of fluorescence spectra

A set of self-written codes in MatLab (MathWorks release 2014a) have been used for pre-processing of all the fluorescence spectra to remove unwanted noise and for the vector normalization. The pre-processed data have been used in producing spectral plots for comparison. A chemometric technique, called principal component analysis (PCA), has been applied on all the spectral data through a built-in routine in MatLab for classification. The principal component analysis (PCA) is the unsupervised chemometric technique that differentiates the samples on the basis of their similarities and differences by plotting them along the orthogonal axes in the form of components called PCs. These PCs are orthogonal vectors, i. e. not corelated to each other. Therefore, when both are plotted against each other as scatter plot, data of both PCs cluster separately.

## Results and discussion

The first type of oil sample, extracted by dissolving crushed canola seeds in hexane in the laboratory will be called chemical method, the second type by pressing canola seeds through the device shown in Fig 1 will be called cold pressed, and the third type taken from commercial canola oil brands will be referred as commercial brands in the discussion.

It is evident from Fig 2 that all commercial brands bear very light color and are almost transparent, while oil extracted by chemical method bears yellow and by cold press bears yellow greenish color. Visually all types of canola oil samples in Fig 2 can be differentiated, but visual inspection cannot ultimately certify the best one. Therefore, in order to evaluate the three types of canola oil samples shown in Fig 2, their emission spectra were recorded by using excitation wavelengths from 280–420 nm and the results are shown in Fig 3, which shows that the emission spectra of commercial brand-2 are entirely different from cold pressed and chemically extracted. The spectra displayed in Fig 3A evidently shows emission bands at 375, 525 and 673 and 725 nm which appeared in canola oil extracted by chemical method. Commercial brand-2 was randomly selected for different experiments described in the text.

The emission band at 375 nm is assigned to vitamin E [21,22], which evidently shows that its intensity decreases with the increase of excitation wavelength and disappears at λ_ex_ of 380 nm. The spectral intensity of emission band at 525 nm, labeled for vitamin E and beta carotenes [19,21,23], increases for λ_ex_ range320-370 nm approaches maximum for λ_ex_ of 370 nm and then decreases and becomes minimum for λ_ex_ of 420 nm. The emission bands at 673 and 725 nm, assigned to chlorophyll derivatives present in canola oil [14,18,24] which were extracted by cold pressed or chemical method. Their intensity increases with the increase of excitation wavelength from 280 nm and becomes maximum at λ_ex_ of 420 nm. Surprisingly, the emission bands at 375, 525, 673 and 725, which have been assigned above were completely absent from the commercial canola brands. However, the emission band centered at 440 nm as shown in Fig 3B appeared in all commercial brands, represent oxidized products of isomers of vitamin E [18,24,25] and fatty acids [11,17]. The band at 440 nm shifted to longer wavelengths for higher excitation wavelengths and it is also reported for Millard-reaction products [26]. Fig 3C shows emission spectra of canola oil extracted by cold press and put in evidence four prominent emission bands centered at 370, 520, 673 and 725 nm and their molecular assignments have been discussed earlier. The emission bands at 375 and 525 nm in Fig 3A for cold pressed canola oil are shifted at 370 and 520 nm in chemically extracted oil. The shifting of spectra may be attributed to matrix effects of oil samples extracted by different methods. However, fluorescence spectra of chlorophylls fluoresce at the same spectral positions as described above. The intensity of emission band centered at 520 nm increases for λ_ex_ range 340–380 nm, it approaches maximum at λ_ex_ of 380 nm and reduces for λ_ex_ range 390–420 nm and become minimum at λ_ex_ of 420 nm. The intensity of emission band at 673 nm increases with the increase of excitation wavelength which means that chlorophylls contents fluoresce more for longer excitation wavelengths and show maximum intensity at λ_ex_ of 420 nm.

**Fig 4:**
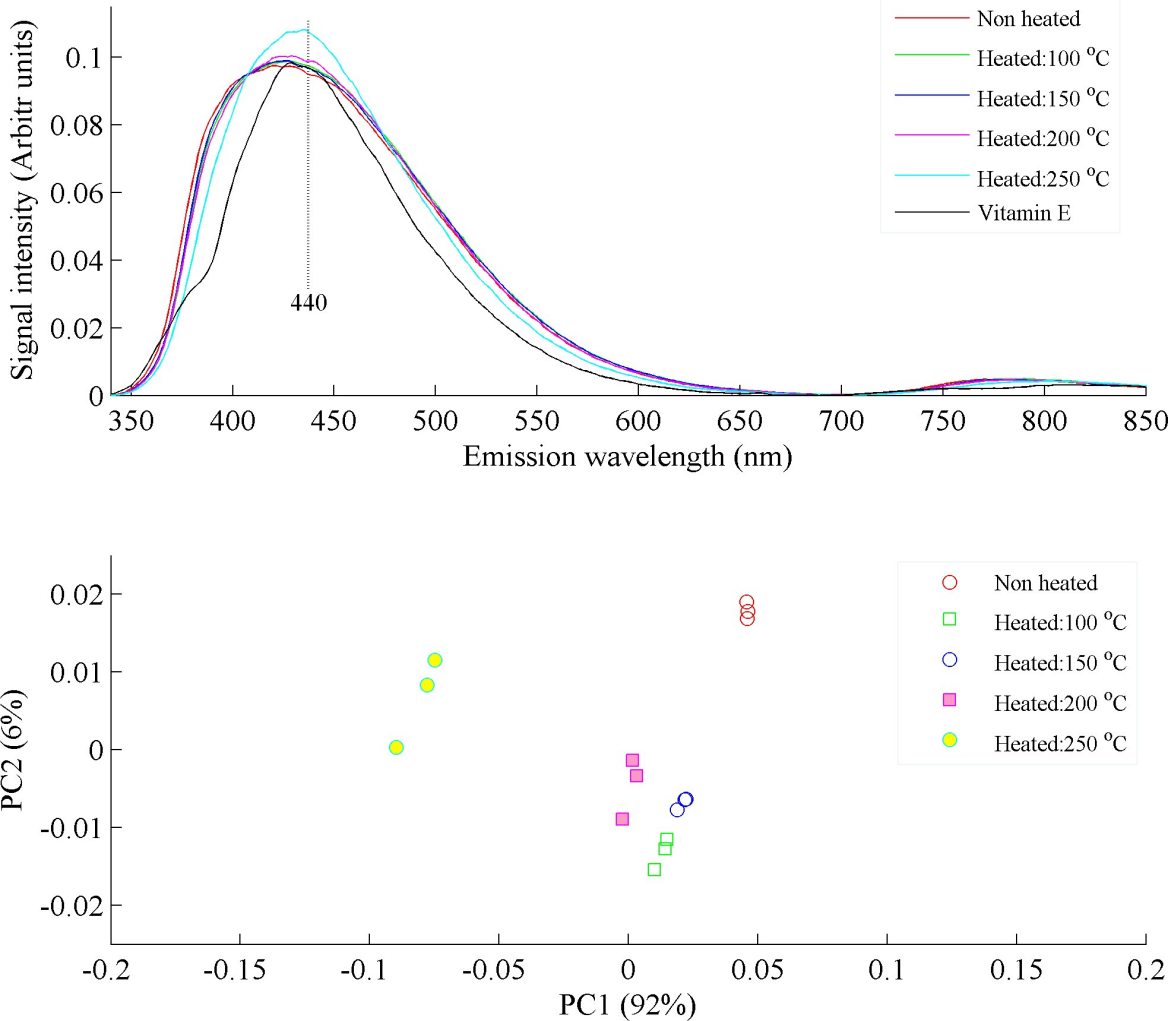
(a) Fluorescence spectra of non-heated and heated commercial brand-2 at different temperatures of 100, 150, 200 and 250°C and vitamin E. (b) PCA scatter plot between PC1 & PC2, which classify the non-heated and heated commercial brand-2, of whom spectra are shown in Fig 4A. PC1 explains 92% and PC2 6% variance in the data.

To investigate the origin of emission band at 440 nm, different aliquots of commercial brand-2 were heated at temperatures of 100, 150, 200 and 250°C for 30 minutes each. The spectra of heated, non-heated and synthesized vitamin E were recorded by using λ_ex_ of 330 nm and are displayed in Fig 4A, which shows that the isomers of vitamin E also fluoresce at 440 nm and this band is also reported for oxidized products of vitamin E [18] but spectral shape of vitamin E is little bit different from others. As reported in the literature, vitamin E fluoresce at 375 and 525 nm [18], this means that the isomers of vitamin E which fluoresce at 440 nm may have different isomers. Regarding the emission band at 440 nm, literature suggested that it is assigned to oxidized products of essential fatty acids and isomers of vitamin E [18,24,25]. It is evident from Fig 4A that there are some little spectral variations in heated commercial oil samples in comparison with non-heated samples and prominent intensity variations can be observed above a temperature of 200 ^o^C. These spectral variations can be better visualized by chemometric analysis, which is shown in Fig 4B. It shows the PCA scatter plot between PC1 (92% variance) and PC2 (6% variance), which classifies the samples on the basis of spectral variations induced due to heating at different temperatures for same time period.

It evidently shows that samples heated up to a temperature of 200°C clustered closely on the positive side of the PC1 axis near to non-heated canola oil samples and samples heated at 250°C clustered on the negative side of the PC1 axis clearly far away from all the rest. However, the trend of clustering of heated samples show that commercial oil samples heated from 100–200°C are not much different from one another which is also evident from the spectra shown in Fig 4A. Therefore, it can be concluded that the commercial brands lose almost all of its natural molecular composition during refining processes and consequently contains only oxidized products which fluoresce. It certainly contains saturated and unsaturated fats but they do not fluoresce but their oxidized products fluoresce. The results in Fig 4 therefore proved that heating causes the evolution of oxidized products which appeared at 440 nm [18,24].

**Fig 5:**
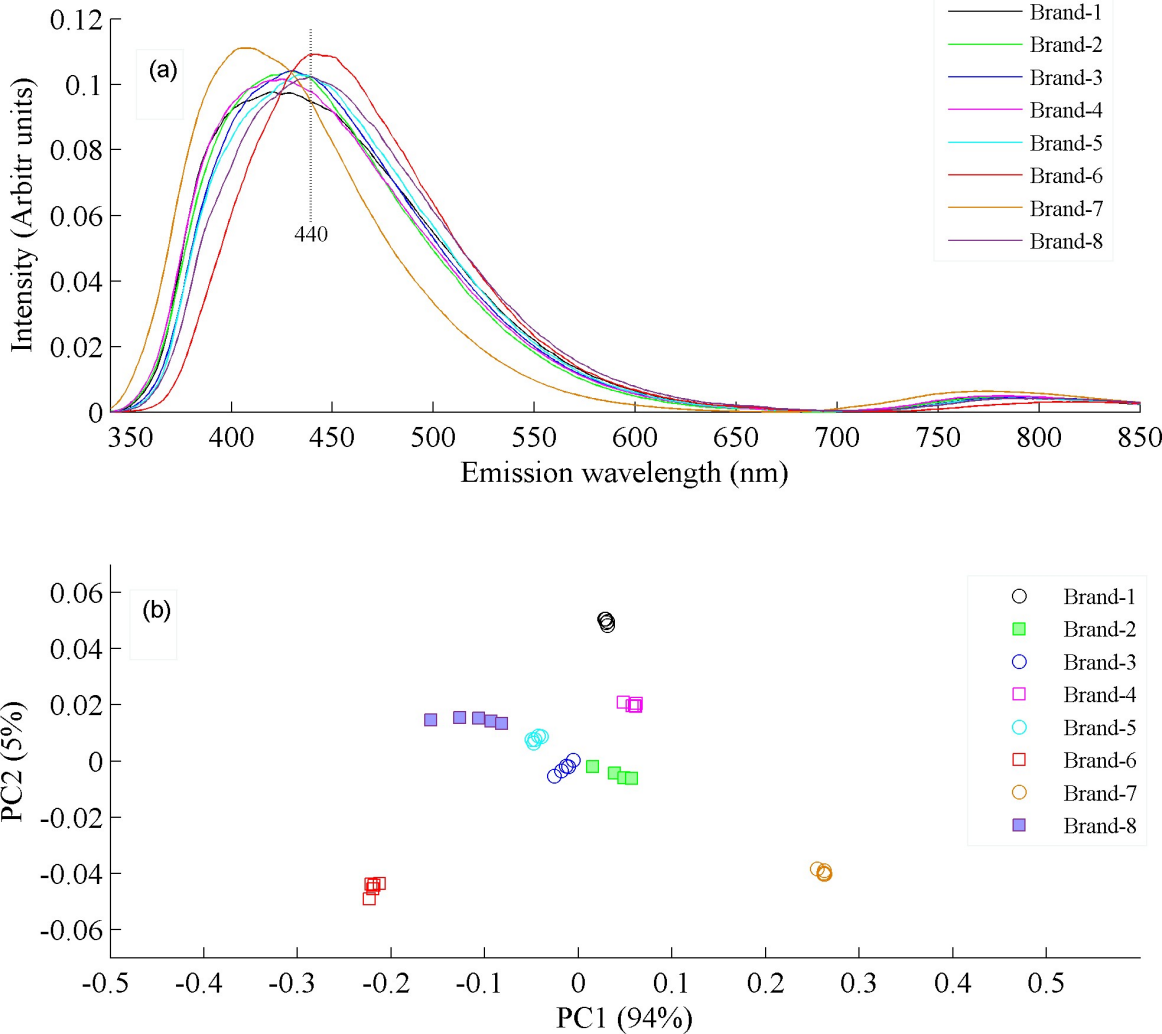
(a) Fluorescence spectra of eight commercial brands available in local markets of Islamabad. (b) PCA scatter plot between PC1 & PC2 which classify eight commercial brands of whom emission spectra is shown in Fig 6A.

In order to further investigate the commercial brand samples in more detail, their fluorescence spectra were acquired with λ_ex_ 330 nm and the results are shown in Fig 5A. All commercial brands show a broad band roughly centered at 440 nm, assigned to oxidized products as described earlier, but the maxima of two bands are at little bit different spectral positions. Fig 5B shows the interspecies PCA classification, where the clustering of commercial brands at different positions in close vicinity illustrated that they contain different concentrations and types of oxidized products, which is also evident from spectra in Fig 5A. Therefore, Fig 5A and 5B confirms that commercially available canola oil samples are treated with almost same processing technologies possibly at very high temperatures that lead to their thermal oxidation and consequently to the destruction of valuable natural ingredients, which have been described earlier.

**Fig 6:**
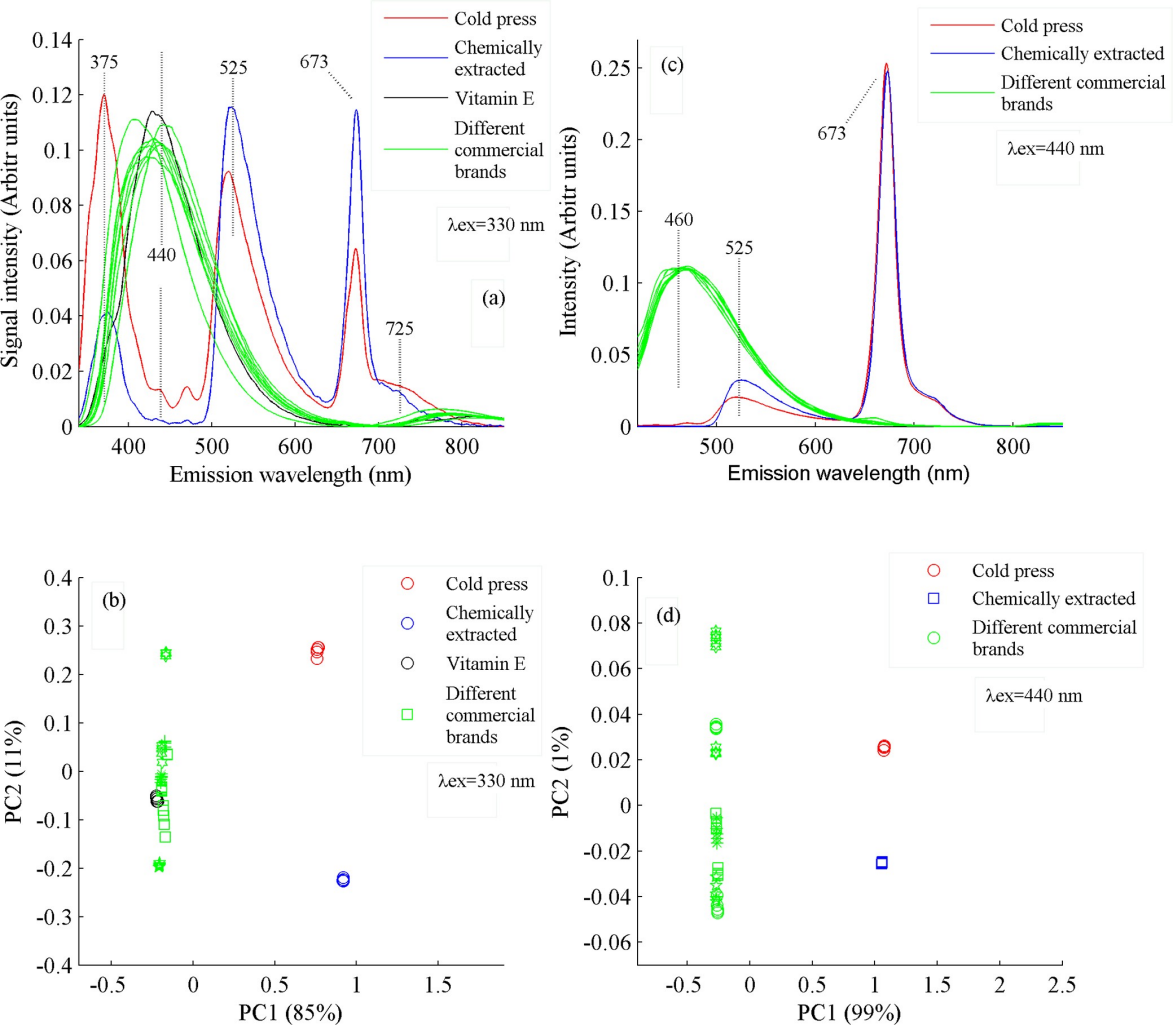
(a) Fluorescence spectra of vitamin E, canola oil samples extracted by cold press, chemical method and eight commercial brands when excited at 330 nm. (b) PCA scatter plot between PC1 & PC2 shows the classification among vitamin E, cold press, chemically extracted and eight commercial brands of canola oil, whom spectra are shown in Fig 6A. (c) Fluorescence spectra of cold pressed, chemically extracted and eight commercial brands of canola oil when excited at 410 nm. (d) PCA scatter plot between PC1 & PC2 shows the classification among cold pressed, chemically extracted and eight commercial brands of canola oil, whom spectra are shown in Fig 6C.

For detailed spectroscopic investigations, three type of oil samples were excited with the two wavelengths of 330 and 410 nm. Both excitation wavelengths were selected because they yield maximum spectral information from the samples. The results are shown in Fig 6, which shows a comparison of fluorescence spectra of vitamin E, commercial canola brands, canola oil extracted chemically and by cold press method when excited by a wavelength of 330 nm. It shows the emission bands at 375, 525, 673 and 725 nm, which are common in oil samples extracted chemically and by cold pressed [21–23] and absent in the eight commercial brands, which consequently leads to the conclusion that commercially available oils do not contain vitamin E, beta carotenoids and chlorophylls. However, they contain oxidized products which came up with emission at 440 nm. The emission band at 375 nm shows high intensity in cold press samples as compared to chemical one. Both bands at 525 and 673 nm show more concentration of vitamin E and beta carotene and chlorophylls contents in chemically extracted canola oil as compared to cold pressed.

The PCA scatter plot in Fig 6B shows that chemically extracted and cold press canola oil samples are clustered towards positive side of PC1 with a clear separation between them, depicting that they have some dissimilar chemical compositions. The difference in the concentration of beta carotene and chlorophyll contents may be the basis for their separation along PC2 axis. It is also evident that commercial canola oil samples along with vitamin E closely clustered in a line along PC2 axis towards negative side of PC1 axis which confirms that all the commercial canola oil brands are different from each other but have similar chemical composition but completely different from cold press and chemically extracted oil. Spectra of vitamin E lies very close to the commercial brands 5 & 8, which means that these brands may have been added by synthesized isomers of vitamin E.

Fig 6C shows a comparison of emission spectra of three types of oil samples excited at λ_ex_ of 410 nm. It shows the intense emission bands at 460, 525 and 673 nm. Bands at 525 and 673 nm are present only in samples extracted chemically and by cold press while emission band at 460 nm is present only in commercial brands. The emission band at 440 nm appeared at the shifted wavelength of 460 nm when excited with λ_ex_ of 410 nm. The band cantered at 460 nm is assigned to oxidized products in cited literature [24]. The band at 673 nm, labelled for chlorophylls shows almost same spectral intensity in chemical and cold press samples. The fluorescence band at 525 nm, assigned to beta carotene and vitamin E shows a little more concentration in chemically extracted oil samples.

Fig 6D shows the PCA scatter plot between PC1 and PC2 which depicted a clear separation of all three types of oil samples. Commercial brands are clustered towards negative side of PC1 axis and show a separating trend along PC2. This trend of commercial oils highlights that they are slightly different from one another, as shown in Fig 6B. While cold pressed and chemically extracted oil samples clustered towards positive side of PC1 clearly separating from each other. This behavior shows that the cold pressed and chemically extracted oil samples show nearly similar composition in comparison with commercial brands, but they are also slightly different from one another.

### Effect of temperature on cold pressed canola oil

**Fig 7:**
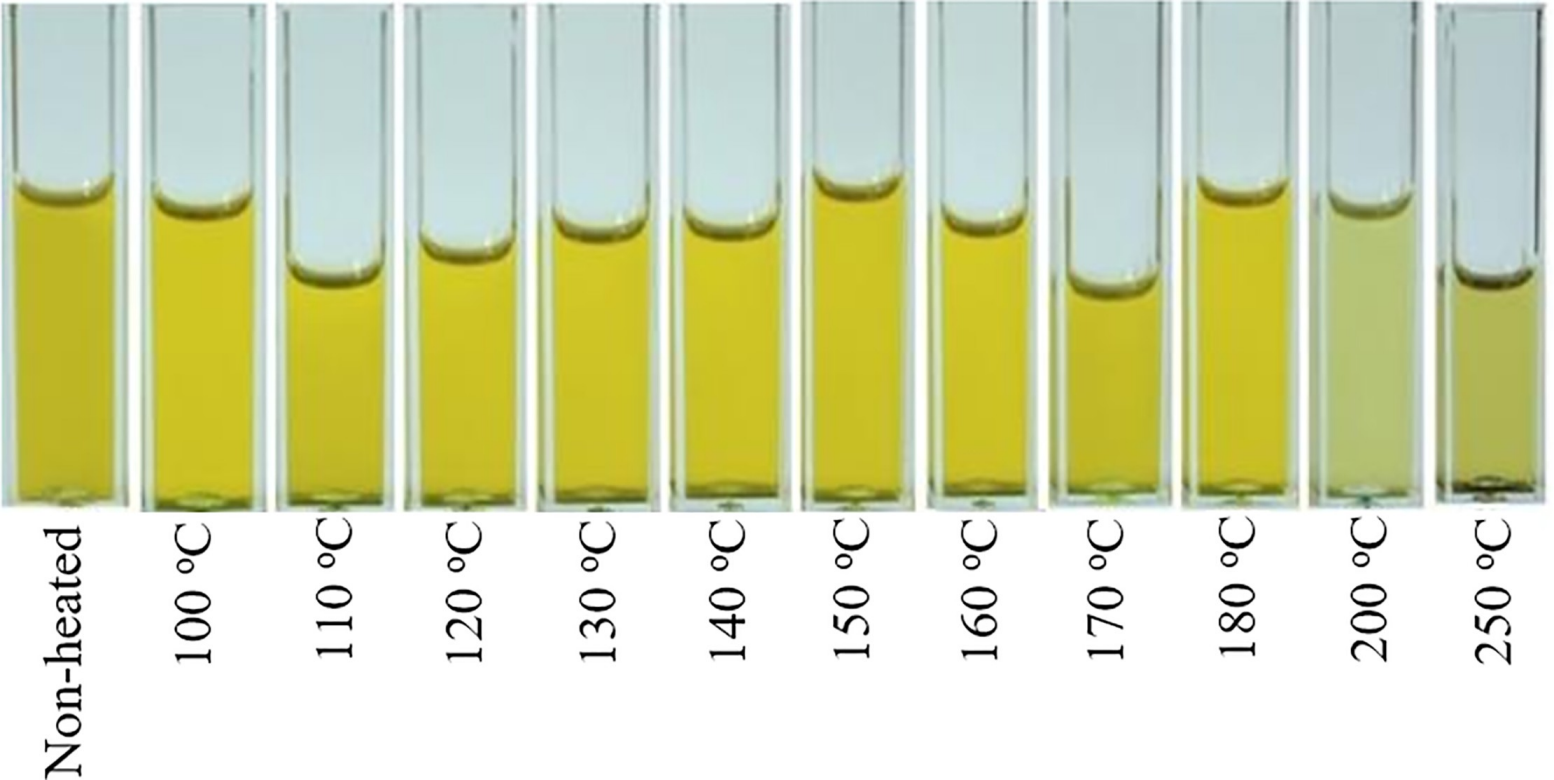
Visual display of non-heated and heated cold pressed canola oil samples at 100, 110, 120, 130, 140, 150, 160, 170, 180, 200 and 250°C.

In order to investigate the temperatures effects on canola oil, different samples of cold press oil have been prepared by heating them at temperatures of 100, 110, 120, 130, 150, 160, 170, 180, 200 and 250°C for 20 minutes. Fig 7 displays the visual effects of temperature on the color of cold pressed canola oils, which shows that up to 180°C visual appearance of oil does not change.

**Fig 8:**
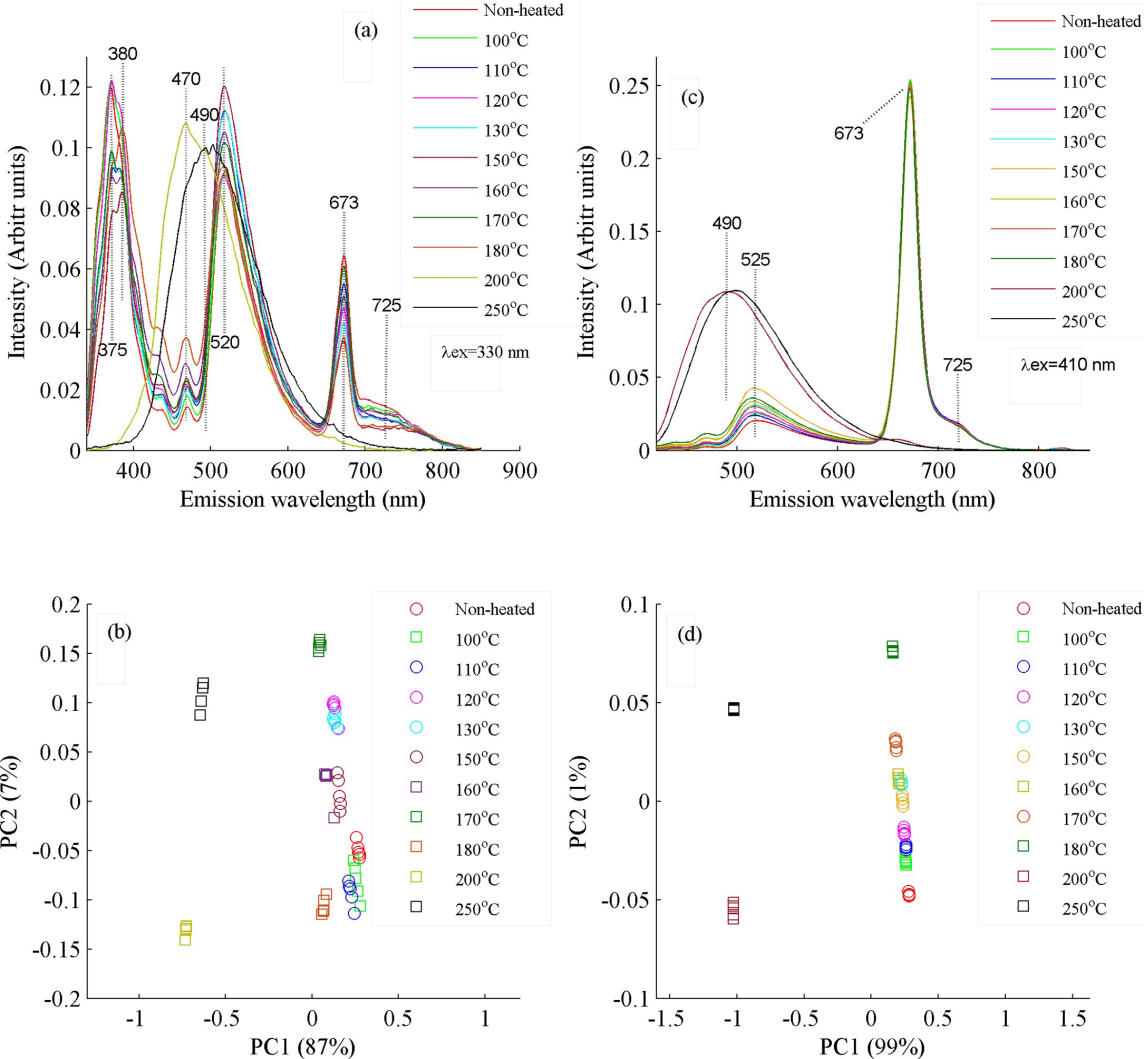
(a) Fluorescence spectra of non-heated and heated cold pressed canola oil samples at 100, 110, 120, 130, 140, 150, 160, 170, 180, 200 and 250°C when excited at wavelength of 330 nm. (b) PCA scatter plot between PC1 and PC2 which shows classification between non-heated and heated cold pressed canola oil samples, whom spectra are shown in Fig 8A. PC1 explains 87% and PC2 7% variances in the data. (c) Emission spectra of non-heated and heated cold press oil samples at 100, 110, 120, 130, 140, 150, 160, 170, 180, 200 and 250°C when excited at wavelength of 410 nm. (d) PCA scatter plot between PC1 and PC2 which shows classification among non-heated and heated cold pressed canola oil samples, whom spectra are shown in Fig 8C. PC1 explains 99% and PC2 1% variances in the data.

However, spectroscopic investigations of all the cold pressed heated oils samples has been carried out using fluorescence spectra recorded with λ_ex_ of 330 and 410 nm where clear spectral variations can be visualized as highlighted in Fig 8.

It is evident from Fig 8A, that up to 180°C, no appreciable spectral changes occur in cold pressed canola oil samples. However, there is a trend of separation in heated samples which can be clearly seen. Above 180°C primary to secondary oxidized products start to appear [27], which reflect themselves as change in band intensity and shape. The intensity of band at 375 nm shows a gradual decrease due to oxidation of essential fatty acids, fat soluble vitamins and carotenoids and this band totally disappears at temperature of 200 and above at 250°C. Emission band at 525 nm, representing vitamin E and beta carotene, shows an increase in its intensity with the rise of temperature above 150°C, possibly due to oxidation of these nutritional ingredients. On the contrary, emission band at 673 nm, representing chlorophylls, shows gradual decrease in its intensity with the rise of temperature. However, from 180 to 200°C, quantity of secondary oxidized products increases and consequently leading to the destruction of vitamin E, beta-carotene and chlorophylls contents which disappeared completely at 200°C, a temperature less than its smoke point [5,20].

Fig 8B shows the PCA scatter plot between PC1 (87% variance) and PC2 (7% variance) which are produced from the emission spectra of heated and non-heated cold pressed oil samples when excited at 330 nm. Cold press oil samples heated up to 180°C, are clustered towards positive side of the PC1 axis while samples heated at 200 and 250°C are clustered towards negative side. It also shows that up to 180°C, there is a small separation trend between non-heated and heated samples which shows that heating influences the chemical composition of oil samples. Above temperature of 180°C, heated samples got separated completely, showing prominent spectral variations due to change in their molecular composition, which is evident from Fig 8A and 8B. Fig 8C shows fluorescence spectra of heated and non-heated cold pressed oil samples using exciting wavelength at 410 nm. Intensity variations can be observed at emission band of 525 and 673 nm as a function of temperature. These two bands totally disappear with the formation of a broad band at 490 nm at temperatures of 200 and 250 ^o^C. The band at 490 nm shows oxidized products and possibly secondary oxidation products which appear at temperatures of 200 and 250°C [27]. Fig 8D shows the PCA scatter plot between the heated and non-heated oil samples of whom spectra are shown in Fig 8C. It shows that along PC1 axis, oil samples heated up to 180°C are clustered towards positive side with minimum separating trend putting in evidence that heating induces minute spectral variations up to this temperature. The samples heated at temperatures of 200 and 250°C cluster towards negative side of PC1 axis and far away from each other which means that they are not only different from each other but also totally different from rest of all samples. The visual look on the displayed heated samples in Fig 7 also reflects the same results. It means that the heating of cold pressed canola oil up to 180°C, does not lose most of its valuable ingredients. Similarly, temperature of 160°C has been recommended for frying of egg in extra virgin olive oil (EVOO) [19], and for deep frying of food stuff in EVOO, temperature of 180°C has been recommended where it does not lose much of its molecular composition [28,29].

**Fig 9:**
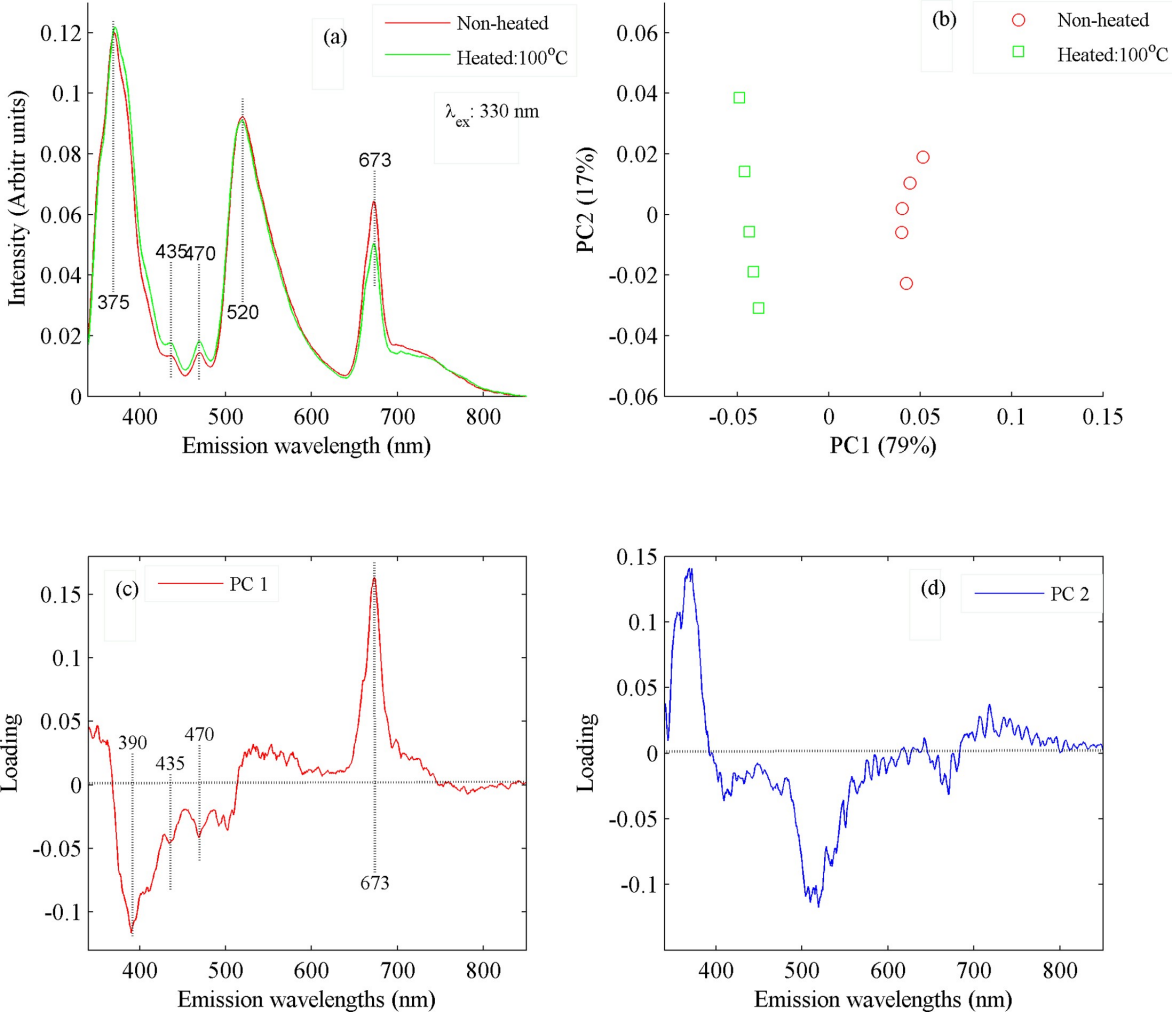
(a) Fluorescence spectra of non-heated and heated cold pressed oil samples at 100°C when excited at 330 nm. (b) The PCA scatter plot between PC1 & PC2 which shows classification between the non-heated and heated cold pressed oil sample at 100 ^o^C, whom spectra are shown in Fig 9A. (c) Loading vector of PC1, which are produced from florescence spectra shown in Fig 9A. (d) Loading vector of PC2, which are produced from florescence spectra shown in Fig 9A.

The PCA classification code is based upon the loading vectors associated with principle components. Loading vectors give the spectroscopic reasoning of classification of data. The PCA code basically calculates the maximum variance between two set of data, therefore, to explain the classification based on loading vectors, the PCA was applied on the spectra of non-heated and heated cold pressed canola oil samples heated at 100°C. The spectra of both samples are shown in Fig 9A, and the PCA scatter plot in Fig 9B and their loading vectors in Fig 9C and 9D). The excitation wavelength of 330 nm has been used for recording emission spectra from non-heated and heated oil sample at 100°C. Fig 9A shows intensity variations in the emission bands at 375, 435, 470, 520 and 673 nm. It evidently shows that heating the cold pressed oil samples, even at comparatively low temperatures, induces small spectral variations. Fig 9B illustrates that the non-heated oil samples are clustered in the positive side and heated at 100 ^o^C in the negative side of the PC1 axis. Same observation is evident from the corresponding loading plots shown in Fig 9C & 9D. It follows that from the spectral features associated with the former are loaded positively, while those of the later are loaded negatively as shown in Fig 9C. Evidently both samples are well separated which is due to maximum variance in PC1, which is 79% and 17% in PC2. However, when it is applied on more than two different samples, it can illustrate the trend of separation between all data based on their spectral dissimilarities.

Loadings of PC1 shows that fluorescence band at 673 nm loaded positively while bands at 390, 435 and 470 loaded negatively which shows that heating at 100 ^o^C induces small changes in the molecular structure of cold pressed oil samples. PC2 in Fig 9D mainly shows noise and negligible variance so it can be neglected. The heated and non-heated cold pressed canola oil samples differ in terms of their molecular composition but these differences are not clearly visible as shown in Fig 7. Fig 9C also shows that heating the cold pressed oil samples start to deteriorate its natural ingredients like vitamin E, beta carotene, essential fatty acids and chlorophylls which reflect the changes at 375, 435, 470 and 520 nm, respectively, and it increases with rise of temperature and heating time. Consequently, it can be inferred from the above discussion that heating of cold pressed canola oil during extraction and refining processes at ultra-high temperatures leaves it with no natural color (beta-carotene & chlorophyll), fatty acids and vitamin E contents which is evident from Fig 2.

Therefore, it can be suggested that cold pressed canola oil is comparatively better than the one extracted chemically in laboratory in terms of their nutritional ingredients. However, both are much better than commercially available canola oil brands. It is, therefore, suggested that extra virgin canola oil extracted through cold press or chemically extracted method is far better to use directly for cooking and frying purposes as it is rich with fat-soluble vitamins, essential fatty acids, beta carotene and chlorophylls.

## Conclusions

Fluorescence spectroscopy along with chemometric analysis have been employed to study the effect of extraction methods on the nutritional values of canola oil. It has been found that canola oil extracted by cold press method is rich with fat soluble vitamins, vitamin E, beta carotene and chlorophylls. Similarly, chemically extracted oil possesses almost same molecular composition as that of cold press but the refining processes destroy its nutritional values. Therefore, if chemically extracted oil is not refined through the processes described in the text then the end users can enjoy its natural ingredients. In addition, it seems difficult to find the origin of commercial brands named as canola oil, as their spectra reflect only oxidized products. Furthermore, it has been investigated that the heating of cold pressed canola oil up to 180°C, does not lose most of its valuable ingredients. Therefore, it can be used safely for cooking of foods where usually temperature remains between 100–115 ^o^C. In addition, present studies suggested that cold pressed canola oil can be used safely without destroying much of its valuable ingredients for frying of egg which needs temperature range 140–160°C and deep frying of meat and fish that needs temperature of oil around 180°C.
